# Alveologenesis: key cellular players and fibroblast growth factor 10 signaling

**DOI:** 10.1186/s40348-016-0045-7

**Published:** 2016-04-21

**Authors:** Cho-Ming Chao, Alena Moiseenko, Klaus-Peter Zimmer, Saverio Bellusci

**Affiliations:** Universities of Giessen and Marburg Lung Center (UGMLC), Excellence Cluster Cardio-Pulmonary System (ECCPS), Member of the German Center for Lung Research (DZL), Department of Internal Medicine II, Aulweg 130, 35392 Giessen, Germany; Division of General Pediatrics and Neonatology, University Children’s Hospital Gießen, Justus-Liebig-University, Gießen, Germany; Institute of Fundamental Medicine and Biology, Kazan (Volga Region) Federal University, Kazan, Russian Federation

**Keywords:** Alveologenesis, Fibroblast growth factor 10 (FGF10), Alveolar epithelial cell type I (AEC I), Alveolar epithelial cell type II (AEC II), Alveolar myofibroblast, Secondary septae

## Abstract

**Background:**

Alveologenesis is the last stage in lung development and is essential for building the gas-exchanging units called alveoli. Despite intensive lung research, the intricate crosstalk between mesenchymal and epithelial cell lineages during alveologenesis is poorly understood. This crosstalk contributes to the formation of the secondary septae, which are key structures of healthy alveoli.

**Conclusions:**

A better understanding of the cellular and molecular processes underlying the formation of the secondary septae is critical for the development of new therapies to protect or regenerate the alveoli. This review summarizes briefly the alveologenesis process in mouse and human. Further, it discusses the current knowledge on the epithelial and mesenchymal progenitor cells during early lung development giving rise to the key cellular players (e.g., alveolar epithelial cell type I, alveolar epithelial cell type II, alveolar myofibroblast, lipofibroblast) involved in alveologenesis. This review focusses mainly on the role of fibroblast growth factor 10 (FGF10), one of the most important signaling molecules during lung development, in epithelial and mesenchymal cell lineage formation.

## Introduction

**Fig. 1:**
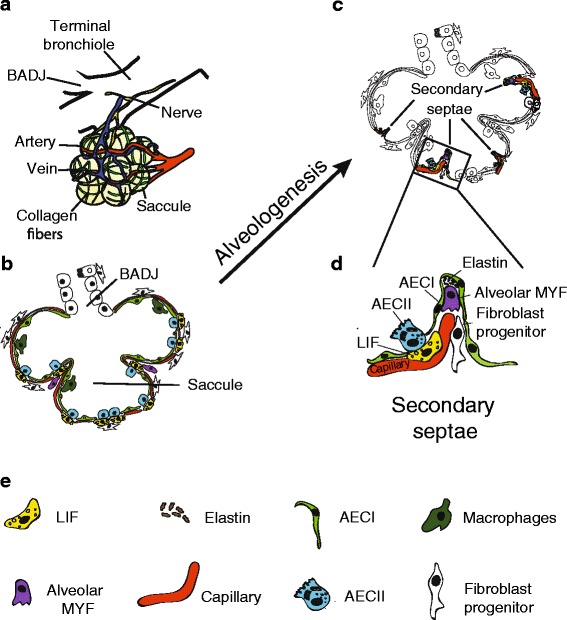
Schematic representation of alveologenesis and cell types involved. **a** During the saccular stage, the lung forms primitive alveoli (saccule) surrounded by collagen fibers, nerves, and blood vessels. **b** The alveolar saccule in the saccular stage is characterized by the presence of AEC I/II, coating the walls of saccule, surfactant production, expansion of capillary tree, production of collagen, and elastin by fibroblasts. **c** During the alveolar stage, the lung undergoes subdividing of sacs by a process called “secondary septation” that will give rise to mature alveoli. **d** Secondary septae starts to appear at the place of elastin deposition, which is produced by alveolar MYF. The septae elongates towards the alveolar sac airspace. Double layer of capillaries become thinner giving rise to a one-layer network for more efficient gas exchange. **e** *AEC I/II* alveolar epithelial cell type I/II, *BADJ* broncho-alveolar duct junction, *LIF* lipofibroblast, *MYF* myofibroblast

Mouse and human lung development consists of four histologically distinguishable stages termed pseudoglandular (mouse, E9.5–E16.5; human, weeks 4–17), canalicular (mouse, E16.5–E17.5; human, weeks 17–26), saccular (mouse, E17.5–P5; human, weeks 26–36), and alveolar (mouse, P5–P30; human, weeks 36–8 years) stages. During the pseudoglandular stage, branching morphogenesis occurs to form the tree-like tubular structure of the lung, which later becomes the conducting airway. At the same time, several mesenchymal and epithelial progenitor cells differentiate into smooth muscle cells, lymphatic cells, endothelial cells, nerve cells, and chondrocytes, as well as basal cells, neuroendocrine cells, ciliated cells, and secretory cells, respectively. Apart from the further branching process of the respiratory bronchioles, one of the main events during the canalicular stage is the formation of a double-layer capillary network. Furthermore, alveolar bipotential progenitor cells give rise to alveolar epithelial cells type I (AEC I) and type II (AEC II) [1], leading to the first primitive respiratory epithelium capable of gas exchange. Another important cell type emerging in canalicular stage is the so-called lipofibroblast (LIF), which is involved in surfactant production through interaction with the AEC II and constitute a potential stem cell niche for AEC II stem cells. The following saccular stage (E17.5-P5) is characterized by alveolar sac formation (primitive alveoli), surfactant production, and the ongoing expansion of the capillary and lymphatic networks (Fig. 1a, b). Through thinning of the mesenchyme due to apoptosis of mesenchymal cells [2], the distance between the blood vessels located in the mesenchyme and the alveolar epithelial surface decreases, thus facilitating oxygen diffusion. Finally, the last stage of lung maturation is the alveolar stage (P5–P30). The lung undergoes a drastic increase in alveolar surface area by subdividing the alveolar sac through a process called the secondary septae formation. This produces the mature respiratory units called alveoli (Fig. 1c). An adult mouse lung consists of about 2.3 Mio. alveoli (alveolar surface area, 82 cm^2^) [3] and in an adult human lung about 300 Mio. alveoli (alveolar surface area, 75 m^2^) [4]. The number of lung lobes and airway generations is also different between a mouse and a human. While the mouse lung is comprised of four lobes on the right side and one lobe on the left side, the human lung develops three lobes on the right and two on the left. The mouse bronchial tree consists of 12 airway generations compared to 23 in the human lung. Last but not least, the probably most interesting difference between mouse and human lung development is that the mouse is born during the saccular stage and the human during the alveolar stage of lung development. Therefore, the hyperoxia-induced lung injury model in the neonatal mouse (bronchopulmonary dysplasia (BPD) mouse model) is a convenient animal model to simulate injurious events in a developmental stage of the lung in which usually preterm infants are born. According to the current knowledge, the alveolar myofibroblast is considered to be the cell type responsible for the secondary septae formation. Furthermore, during alveolar stage, the lung vasculature, which still consists of a double layer of capillaries, becomes a single capillary network via a process called microvascular maturation in order to allow more efficient oxygen intake (Fig. 1d). In contrast to the above-mentioned classification of lung development into four stages, it has recently been suggested to classify lung development according to two distinct developmental processes: (1) the branching morphogenesis program (E9.5–E16.5) and (2) the alveolar epithelial differentiation program (E16.5–P30). During the branching morphogenesis program, the distal epithelial progenitors give rise to several differentiated epithelial cell types of the conducting airway [5]. During the alveolar epithelial differentiation program, the epithelial progenitors give rise to the AEC I/AEC II cells [1].

This review will summarize the current understanding of lung epithelial and mesenchymal cell lineage formation during development, while focusing on key cellular players (Table 1) and the fibroblast growth factor 10 (FGF10) signaling pathway involved in alveologenesis. We refer to other outstanding reviews for more detailed information on lung development [6].

**Table 1:** Overview of cells relevant for alveologenesis

Name of cell	Name of progenitor cell	Localization of cell	Function of cell for alveologenesis	Interaction with other cells
Alveolar epithelial cell type I (AEC I)	Bipotent progenitor (*Sftpc+*, *Pdpn+*) [1]	Epithelium	Providing the majority of alveolar surface area	AEC II (during regeneration) [16]
Alveolar epithelial cell type II (AEC II)	Bipotent progenitor (*Sftpc+*, *Pdpn+*) [1]	Epithelium, close proximity to lipofibroblast	Surfactant production, transdifferentiation to AEC I after lung injury [16]	Formation and maintenance of Lipofibroblast via Pthrp (parathyroid hormone-related protein)/Pparg (peroxisome proliferator-activated receptor gamma) signaling pathway [35, 37]
Alveolar myofibroblast	Alveolar myofibroblast progenitor; *Pdgfra* + LIF (to be validated) [25]	Mesenchyme, Tip of growing secondary septae	Deposition of Elastin in the apex of secondary septae and secondary septae formation [30, 55]	Not known
Lipofibroblast	*Fgf10+* mesenchymal cell [32, 33]	Mesenchyme, close proximity to AEC II	Secretion of triglycerides and leptin for AEC II [37] postnatal niche for AEC II [34]	AEC II [35, 37], epithelium (Pdgfa) [31]
Endothelial cell	Endothelial progenitor cell (hemangioblasts)	Subepithelial mesenchyme (SEM)	Angio-/vasculogenesis important for alveologenesis [47]	Epithelium [43, 45]

## Epithelial cell lineage formation and FGF10 signaling

Beginning at the pseudoglandular stage of embryonic lung development, a complex interaction amongst the following three morphologically distinguishable compartments of the lung occurs: mesothelium (most distal/outer layer), mesenchyme (middle layer subdivided into submesothelial mesenchyme (SMM), and subepithelial mesenchyme (SEM)) and epithelium (proximal/inner layer). Additionally, the endothelium arises within the mesenchyme from E10 onwards. Through an elaborate signaling network, these different compartments interact with each other via a process called induction to specify the differentiation of the adjacent tissue in a certain direction [7]. This process controls the proliferation, amplification, differentiation, and migration of diverse epithelial and mesenchymal progenitor cells along the proximal-distal axis of the lung. In the following section, the present knowledge on epithelial cell lineage formation during lung development—with emphasis on the AEC I and AEC II cells—will be reviewed.

The first multipotent epithelial progenitor cell detected at the distal tip of the embryonic lung at E10.5 expressed *SRY (sex determining region Y)-box 9* (*Sox9*) as well as the *inhibitor of differentiation 2* (*Id2*). Using a lineage tracing approach (*Id2*^*Cre-ERT2*^ knock-in mouse line), Rawlins and colleagues demonstrated that *Id2+* progenitor cells labeled at the pseudoglandular stage give rise to all the epithelial cell types of the lung [8]. By contrast, labeling of *Id2+* cells during the canalicular stage captured only the differentiated cells along the alveolar lineage, the AEC I and II cells. These findings suggest that *Id2* during the pseudoglandular stage is a marker for the multipotent epithelial progenitor cells, which initially differentiate into bronchiolar progenitors (*Sox2+*). The bronchiolar progenitor cells give rise to club cells, ciliated cells, goblet cells, and neuroendocrine cells, all of which populate the conducting airway epithelium. Interestingly, lineage tracing using the calcitonin gene-related peptide (*Cgrp*) promoter to target the neuroendocrine cells during lung development showed that Cgrp+ cells labeled at E12.5 can also give rise to AEC I and AEC II cells in the adult lung [9]. The exact relationship between Cgrp+ cells (which include mostly the neuroendocrine cells) and other lung cell types during normal development and homeostasis is still under investigation. As development proceeds, multipotent epithelial progenitor cells give rise during the canalicular stage to alveolar/bipotent progenitors (*Id2+*, *Sox9+*, *Pdpn+*, *Sftpc+*), which contribute to the AEC I and AEC II cells. The cellular and molecular mechanisms regulating these different developmental decisions are still unclear. In particular, what is controlling the differentiation of the multipotent progenitors (*Id2+, Sox9+*) into alveolar/bipotent progenitors (*Id2+, Sox9+, Podoplanin+, Sftpc+*) is so far unknown. Chang and colleagues showed that the epithelial overexpression of small GTPase Kras inhibited differentiation towards the AEC I/AEC II cell lineage but promoted the branching program [5]. In addition, it has been demonstrated that ubiquitous expression of *Fgf10* from the beginning of the pseudoglandular stage prevents the differentiation of multipotent epithelial progenitor cells into *Sox2+* bronchiolar progenitor cells [10]. Additionally, the ubiquitous expression of *Fgf10* at later time points during the pseudoglandular stage, which allows the formation of *Sox2+* bronchiolar progenitor cells, prevented the differentiation of these bronchiolar progenitors into ciliated cells, directing them instead towards the *p63+* basal cell lineage [10]. Using the *Ccsp-rtTA;tet(O)Cre* mouse line to permanently activate green fluorescent protein (GFP) reporter expression in the context of a lineage tracing experiment, it was reported that club cells (secretoglobin, family1A, member 1 (*Scgb1a1+*)) give rise to club and goblet cells, whereas neuroendocrine cells remained unlabelled [11].

## The role of FGF10 in the formation and maintenance of the alveolar lineage is still elusive

Interestingly, bipotent (*Sftpc+, Pdpn+*) progenitor cells, which give rise to AEC I and AEC II, express high levels of *Etv5*, a downstream target of FGF signaling, as well as the gene encoding the FGF10 receptor, *Fgfr2b* [1]. Due to the lung agenesis phenotype displayed by *Fgf10* [12] or *Fgfr2b* null embryos [13], the role of FGF10/FGFR2b signaling in the formation of the alveolar lineage has been suggested mostly from partial loss or gain of function approaches. *Fgf10* is one of the most important developmental genes expressed in the submesothelial mesenchyme of the developing lung from the onset of organogenesis. It encodes a secreted diffusible protein, which acts in a paracrine fashion mainly through the epithelial receptor FGFR2b [14] during the pseudoglandular stage of lung development (embryonic day E9.5 through E16.5 in mice). FGF10 plays a crucial role in controlling epithelial morphogenesis, a process forming a stereotypic set of epithelial tubes organized as a tree and which are the precursors of the conducting airways. In vitro experiments suggest that FGF10 acts primarily on the epithelium by promoting chemotaxis rather than proliferation [15]. In addition, during the pseudoglandular stage, FGF10 has been shown to maintain the undifferentiated status of the SOX9+/ID2+ cells in the distal epithelium [10]. Based on single-cell transcriptomics studies of the epithelium during lung development, the alveolar progenitors have been proposed to represent a progenitor population-baptized “bipotent progenitor cells” [1]. These bipotent epithelial progenitor cells can differentiate into either AEC I or AEC II cells, but what is controlling their differentiation is still unknown. Desai and colleagues demonstrated that postnatally new AEC I cells derive from mature AEC II cells produce expanding clonal foci of alveolar renewal. This stem cell function of the AEC II is induced by injury of the alveolar epithelium [16]. Interestingly, FGF10 is being secreted by LIFs (see below mesenchymal cell lineage formation and FGF10 signaling). LIFs are located in close proximity to AEC II. It is tempting to speculate that FGF10 secreted by the LIFs is essential for maintaining the AEC II stem cells, but this remains to be tested experimentally in mice via the deletion of the main receptor *Fgfr2b* in conjunction with lineage tracing in the mature AEC II cells. A gene signature characteristic of AEC I and AEC II has been reported. The bipotent progenitor cells exhibit both signatures. Unpublished data indicate that FGF10 could play an important role in directing the differentiation of the bipotent progenitor cells towards the AEC II lineage (Chao C. M. and Bellusci S., in preparation), and also for the differentiation of the vascular system and the formation of the alveolar myofibroblasts, a key cell type in the secondary septae. Consistent with this possibility, we previously reported that use of an *Fgf10* hypomorph allele, which constitutively reduces *Fgf10* expression (to around 20 % of the normal level observed in WT), leads to impaired alveolar lineage formation with a significant reduction of surfactant proteins [17]. During the pseudoglandular stage, FGF10 also indirectly orchestrates the development of the adjacent mesenchyme, which contains the progenitors for a number of important lung mesenchymal cell types, including the endothelium, the vascular and airway smooth muscle cells, the alveolar myofibroblasts, the lipofibroblasts, the interstitial fibroblasts, and the nerve cells [7, 18]. More detailed information about the alveolar myofibroblasts and lipofibroblasts will be given in the next section. Such impact on the mesenchyme is unsurprising, as FGF10 signaling is embedded in an interactive signaling network comprising major pathways such as retinoid acid, Sonic hedgehog, bone morphogenetic protein 4, transforming growth factor ß-1, WNT, platelet-derived growth factor, and vascular endothelial growth factor. In addition to its developmental role, FGF10 is a major player in the regeneration of the lung after injury. Upon bleomycin-mediated lung injury in mice, fibrosis development is thought to occur primarily via alveolar epithelial cell damages and pulmonary inflammation. In this model, *Fgf10* overexpression demonstrated a protective and therapeutic effect by increasing fibrosis resolution. The proposed mechanism is FGF10-mediated AEC II survival. FGF10 is also involved in the regeneration of the bronchial lung epithelium after naphthalene injury [19, 20].

## Mesenchymal cell lineage formation and FGF10 signaling

Lung mesenchymal cells such as smooth muscle cells (SMCs), endothelial cells, nerve cells, lipofibroblasts, alveolar myofibroblasts, lymphatic cells, and others play a very important role in lung development and homeostasis. During the alveolar stage of lung development, two mesenchymal lineages are prevalent: the alveolar myofibroblasts (MYF) and the lipofibroblasts (LIF).

## Alveolar myofibroblasts (MYF) are believed to play a key role in the process of alveologenesis

Alveolar MYF are present in the lung during the postnatal alveolar stage, being located at the tip of forming the secondary septae. They form elongated cytoplasmic protrusions to establish a continuous loop around the nascent septal cup. These loops are interconnected, giving rise to a “fishnet”-like α-smooth muscle actin network, with strings of the fishnet underlying future alveolar ridges [21]. Alveolar MYF are defined by expression of alpha-smooth muscle actin (*a-SMA or Acta2*) and production of extracellular matrix fibers, such as elastin and collagen. Deposition of elastin, which allows the alveoli to stretch during the inhalation process, is an essential process for septation [22]. After birth, elastin forms a matrix, serving as a scaffold on which alveolar MYF adheres and marking sites of the secondary septae. *Elastin*-knock-out mice failed to form the secondary septae, which led to an emphysematous-like phenotype and the mutant mice dying within a few days after birth, prior to the onset of alveologenesis [23].

It is believed that alveolar MYF are derived from *Pdgf receptor alpha* (*Pdgfra*)-positive cells. During alveologenesis, PDGFRa-positive cells are present at the tips of the forming secondary septae. In *Pdgfa*-null mice (PDGFa is the ligand of PDGFRa), PDGFRa-positive cells are no longer present in the lung and this is associated with an impaired secondary septae formation [24]. The origin of the alveolar myofibroblasts is still controversial. However, it appears that FGFR2b ligands are critical for their formation. Indeed, *Fgf10* hypomorphic lungs display enlarged respiratory airways at birth characterized by the absence of smooth muscle actin-positive myofibroblasts [17]. Furthermore, inhibition of all FGFR2b ligands using a dominant negative approach with expression of a soluble form of FGFR2b (*Spc-rtTA/+*; *tet(O)solFgfr2b/+*) from E14.5 to E18.5 disrupts alveologenesis. In this context, defective secondary septae formation can be corrected by retinoic acid administration. However, re-expression of the *dominant negative sFgfr2b* to inhibit FGFR2b ligands blocks the retinoic acid-induced regeneration process, indicating that FGFR2b ligands expressed in the postnatal lung are also critical for the formation of the secondary septae [25]. Additionally, it was shown that in a model of re-alveolarization after pneumonectomy, FGFR2b ligands are required for alveolar MYF formation during regeneration [26]. This study also proposed that alveolar MYF arise from *Pdgfra*-positive LIF and that FGFR2b ligands (likely FGF10) are (is) involved in the differentiation of *Pdgfra*-positive cells into alveolar MYF. Altogether, these data indicate that one or several FGFR2b ligands, in particular FGF10, are/is directly responsible for the formation of the alveolar myofibroblasts. However, it is still unclear if this results from a direct effect of FGF10 on the alveolar myofibroblast progenitors or if this effect is indirect via the action of FGF10 on the AEC II cells, which are also a major component of the forming alveoli. The use of specific driver lines to target the alveolar myofibroblast progenitors will be instrumental to follow their fate in the context of lung injury or regeneration. Interestingly, increased levels of FGF signaling in the lung mesenchyme during development leads to absence of alveolar MYF at birth and reduced elastin deposition [27]. FGF signaling is therefore also capable of repressing the differentiation of the alveolar MYF progenitors. It is possible that different FGF ligands, acting via either the c- or the b-isoform of FGF receptors, either prevent the differentiation of the alveolar MYF progenitors allowing their proliferation or trigger their differentiation. Further investigation will be required to test this possibility. Recently, Branchfield and colleagues suggested that *Pdgfra*-positive cells differentiate into MYF during alveologenesis and later adopt a lipofibroblast fate [21]. However, as these experiments were not based on lineage tracing approaches, these conclusions are still debatable. It has also been suggested from in vitro studies that sonic hedgehog (SHH) signaling drives migration of lung alveolar myofibroblasts towards the sites of secondary septation [28]. *Gli1* is known to be a downstream target of SHH signaling. By using a *Gli1-Cre*^*ERT2*^ mouse line, which targets cells that are responding to SHH signaling, Li and colleagues demonstrated that *Gli1+* cells labeled during early embryonic development (E10.5–E11.5) served as progenitors for alveolar MYF. This process also depended on WNT signaling [29].

## Lipofibroblast is a subset of fibroblasts characterized by the presence of lipid droplets

These cells are abundant in the late stages of lung development and postnatally and are located in close proximity to AEC II [30]. LIF serve as a source of lipids, which are transported to AEC II for surfactant production [31]. Recently, it was shown that *Fgf10*-expressing cells give rise to a subset of the LIF lineage during development [32]. Additionally, in postnatal lungs, a significant proportion of LIF express *Fgf10* and FGF10 appears to contribute to their formation [33]. We hypothesize that FGF10 secreted by the LIFs is essential for maintaining the AEC II stem cells, but this remains to be tested. It was shown that *Pdgfra+* cells/LIF constitute a stem cell niche for AEC II stem cells [34]. LIF and AEC II co-cultured in Matrigel form alveolosphere-like structures. It has been proposed that AEC II signal to LIF via parathyroid hormone-related protein (PTHRP), which then activates the peroxisome proliferator-activated receptor gamma (PPARg) pathway. Activation of the PPARg pathway leads to expression of adipose differentiation-related protein (*Adrp* or *perilipin 2*, *Plin2*) by LIF. ADRP is necessary for lipid trafficking and regulation of surfactant production. In addition to triglycerides, LIF also secretes leptin and retinoic acid, both of which are necessary for surfactant production and alveolar septation [35–37].

During the early pseudoglandular stage (E10.0), the lung vasculature starts to form by processes called angiogenesis and vasculogenesis. During angiogenesis, the capillaries form by sprouting from pre-existing endothelial cells, whereas vasculogenesis is characterized by migration and differentiation of endothelial progenitor cells (or hemangioblasts) in the distal mesenchyme to form new blood vessels. Several studies have confirmed that endothelial progenitors are located in the SEM and expressed vascular endothelial growth factor receptor 2 (*Vegfr2* or *Flk-1*) as an early marker [38]. However, the origin of the endothelial progenitors is still not clear. Most of the endothelial progenitors seem to arise from *Wnt2+*, *Gli1+*, and *Isl1+* cells (coming from the second heart field) [39] as well as *Pdgfrb+* cells [40] and mesothelial cells [41]. Recently, our group demonstrated that *Fgf10+* cells give rise to a subset of progenitors for the vascular smooth muscle cells [32]. Increasing evidence gained from in vitro recombination studies (co-culture of epithelium and mesenchyme and mesenchyme alone) as well as in vivo animal models showed that endothelial-epithelial tissue interaction is essential for branching morphogenesis as well as alveologenesis. However, this is still controversial as it has recently been shown in vitro that the endothelial cells are not required for the branching of the lung epithelium. In E12.5 lung explants, the lung epithelium was able to branch despite blockade of vascular endothelial growth factor (VEGF) signaling with three different VEGF receptor inhibitors (SU5416, Ki8751, and KRN633) [42]. On the other side, supporting a role of the endothelium in the branching of the lung epithelium, Lazarus and colleagues demonstrated that the epithelial branching process was interrupted upon an in vivo inducible decoy VEGFR1 receptor expression (to block VEGFR1 ligands such as VEGFa) [43]. The authors also found that *Spry2* is upregulated in the epithelium upon inhibition of VEGFR1-mediated signaling, suggesting an inhibition of FGF signaling (SPRY2 is an inhibitor of FGF10) which is essential for branching morphogenesis [43]. Consistent with this, FGF10 leads to the upregulation of *Vegf* in the distal epithelium [44]. We previously published that embryonic lung explants treated with recombinant VEGFa led to upregulation of *Vegfr2* in the mesenchyme and branching of the epithelium [45]. Whether this effect of VEGFa on epithelial branching is direct or indirect needs to be investigated. Additional strong evidence came from the platelet endothelial cell adhesion molecule (*Pecam1*)-deficient mice that failed to form endothelial cells and displayed alveolar simplification [46]. Consistent with the findings that VEGFa is downregulated in preterm infants with BPD, Thebaud and colleagues showed in a rat model of BPD that adenoviral administration of VEGF improved alveolar architecture [47]. However, the use of VEGF alone as a treatment may be limited as capillary leakage is also observed upon VEGF treatment [47]. Another confirmation for the link between the lung vasculature and alveologenesis has been demonstrated convincingly by induced endothelium-specific deletion of *Vegfr2* and *Fgfr1* in a pneumonectomy mouse model leading to impaired compensatory lung growth [48]. Based on these data, the current consensus is that vascular growth is a driving force for alveologenesis.

## FGF10 in human lung diseases

Heterozygous mutations in the human *FGF10* or *FGFR2B* gene result in aplasia of lacrimal and salivary glands (ALSG) and lacrimo-auriculo-dento-digital syndrome (LADD), respectively [49, 50]. While babies with ALSG or LADD do not display apparent lung defects, adult patients with heterozygous loss of function of *FGF10* exhibit a significant decrease in inspiratory vital capacity (IVC), forced expiratory volume in one second (FEV1), and FEV1/IVC quota compared to non-carrier siblings and predicted reference values [51]. These data are consistent with chronic obstructive pulmonary disease. Based on our recent data gained from *Fgf10* heterozygous (*Fgf10+/−*) mice (Chao C. M. and Bellusci S., data not published), we hypothesize that these patients acquired a lung phenotype which might be due to quantitative and qualitative congenital defects of the AEC I and AEC II cells emerging during alveolar cell lineage formation and leading postnatally to abnormal epithelial repair processes after injury. In humans, exposure to inflammation is known to increase the risk for developing BPD [52]. Consistent with this fact, it has been demonstrated that interactions between nuclear factor “kappa-light-chain-enhancer” of activated B cells (NF-κB), specificity protein 1 (SP1), and SP3 led to inhibition of *Fgf10* expression [53]. *Fgf10* inhibition is mediated by Toll-like receptor 2 and 4 (TLR2 or TLR4) activation, and decreased *FGF10* concentration was found in lung samples from children suffering from BPD [54]. These data provided clues to molecular mechanisms linking inflammatory signaling to this important developmental gene, which might play a role in BPD pathogenesis.

## Summary

In view of the numerous lung diseases characterized by a lack and/or destruction of alveoli (e.g., BPD, chronic obstructive pulmonary disease (COPD)), the fundamental understanding of the alveologenesis process with its coordinated cellular interactions and intricate signaling network occurring between epithelial, mesenchymal, and endothelial is essential. For this purpose, developmental biologists have been working extensively to unravel the molecular and cellular bases of mouse lung development, both pre- and postnatally. In the future, the pneumonectomy mouse model combined with genetically modified mouse lines, lineage tracing approaches, and single-cell transcriptomic analyses will be powerful tools to shed new lights on the regenerative aspects associated with de novo alveologenesis. Such knowledge is critical to develop innovative therapies to treat lung diseases.

## Abbreviations

Adrp: adipose differentiation-related protein
AEC I: alveolar epithelial cell type I
AEC II: alveolar epithelial cell type II
ALSG: aplasia of lacrimal and salivary glands
BADJ: broncho-alveolar duct junction
Bmp 4: bone morphogenetic protein 4
BPD: bronchopulmonary dysplasia
Ccsp: club cell secretary protein
COPD: chronic obstructive pulmonary disease
E: embryonic
ETV5: Ets variant 5
FEV1: forced expiratory volume in one second
Fgf10: fibroblast growth factor 10
Fgfr: fibroblast growth factor receptor
GFP: green fluorescent protein
Gli1: glioma-associated oncogene 1
Id2: inhibitor of differentiation 2
Isl1: insulin gene enhancer protein ISL-1
IVC: inspiratory vital capacity
LADD: lacrimo-auriculo-dento-digital syndrome
LIF: lipofibroblast
MYF: myofibroblast
NF-κB: nuclear factor “kappa-light-chain-enhancer” of activated B cells
P: postnatal
Pdgf: platelet-derived growth factor
Pdpn: podoplanin
Pecam1: platelet endothelial cell adhesion molecule
Pparg: peroxisome proliferator-activated receptor gamma
Pthrp: parathyroid hormone-related protein
RA: retinoid acid
Scgb1a1: secretoglobin, family1A, member 1
SEM: subepithelial mesenchyme
Sftpc or Spc: surfactant protein C
SHH: sonic hedgehog
SMM: submesothelial mesenchyme
Sox 9: SRY (sex determining region Y)-box 9
Sp1: specificity protein 1
Spry2: Sprouty 2
Tgf-ß1: transforming growth factor ß-1
TLR: Toll-like receptor
Vegf: vascular endothelial growth factor
Vegfr: vascular endothelial growth factor receptor
Wnt: wingless and int
α-SMA: α-smooth muscle actin

## References

[1] Treutlein B, Brownfield DG, Wu AR, Neff NF, Mantalas GL, Espinoza FH, Desai TJ, Krasnow MA, Quake SR (2014). Reconstructing lineage hierarchies of the distal lung epithelium using single-cell RNA-seq. Nature.

[2] Kresch MJ, Christian C, Wu F, Hussain N (1998). Ontogeny of apoptosis during lung development. Pediatr Res.

[3] Knust J, Ochs M, Gundersen HJ, Nyengaard JR (2009). Stereological estimates of alveolar number and size and capillary length and surface area in mice lungs. Anat Rec (Hoboken).

[4] Ochs M, Nyengaard JR, Jung A, Knudsen L, Voigt M, Wahlers T, Richter J, Gundersen HJ (2004). The number of alveoli in the human lung. Am J Respir Crit Care Med.

[5] Chang DR, Martinez Alanis D, Miller RK, Ji H, Akiyama H, McCrea PD, Chen J (2013). Lung epithelial branching program antagonizes alveolar differentiation. Proc Natl Acad Sci U S A.

[6] Warburton D, El-Hashash A, Carraro G, Tiozzo C, Sala F, Rogers O, De Langhe S, Kemp PJ, Riccardi D, Torday J, Bellusci S, Shi W, Lubkin SR, Jesudason E (2010). Lung organogenesis. Curr Top Dev Biol.

[7] El Agha E, Bellusci S (2014). Walking along the fibroblast growth factor 10 route: a key pathway to understand the control and regulation of epithelial and mesenchymal cell-lineage formation during lung development and repair after injury. Scientifica.

[8] Rawlins EL, Clark CP, Xue Y, Hogan BL (2009). The Id2+ distal tip lung epithelium contains individual multipotent embryonic progenitor cells. Development.

[9] Song H, Yao E, Lin C, Gacayan R, Chen MH, Chuang PT (2012). Functional characterization of pulmonary neuroendocrine cells in lung development, injury, and tumorigenesis. Proc Natl Acad Sci U S A.

[10] Volckaert T, Campbell A, Dill E, Li C, Minoo P, De Langhe S (2013). Localized Fgf10 expression is not required for lung branching morphogenesis but prevents differentiation of epithelial progenitors. Development.

[11] Perl AK, Wert SE, Loudy DE, Shan Z, Blair PA, Whitsett JA (2005). Conditional recombination reveals distinct subsets of epithelial cells in trachea, bronchi, and alveoli. Am J Respir Cell Mol Biol.

[12] Sekine K, Ohuchi H, Fujiwara M, Yamasaki M, Yoshizawa T, Sato T, Yagishita N, Matsui D, Koga Y, Itoh N, Kato S (1999). Fgf10 is essential for limb and lung formation. Nat Genet.

[13] De Moerlooze L, Spencer-Dene B, Revest JM, Hajihosseini M, Rosewell I, Dickson C (2000). An important role for the IIIb isoform of fibroblast growth factor receptor 2 (FGFR2) in mesenchymal-epithelial signalling during mouse organogenesis. Development.

[14] Ohuchi H, Hori Y, Yamasaki M, Harada H, Sekine K, Kato S, Itoh N (2000). FGF10 acts as a major ligand for FGF receptor 2 IIIb in mouse multi-organ development. Biochem Biophys Res Commun.

[15] Bellusci S, Grindley J, Emoto H, Itoh N, Hogan BL (1997). Fibroblast growth factor 10 (FGF10) and branching morphogenesis in the embryonic mouse lung. Development.

[16] Desai TJ, Brownfield DG, Krasnow MA (2014). Alveolar progenitor and stem cells in lung development, renewal and cancer. Nature.

[17] Ramasamy SK, Mailleux AA, Gupte VV, Mata F, Sala FG, Veltmaat JM, Del Moral PM, De Langhe S, Parsa S, Kelly LK, Kelly R, Shia W, Keshet E, Minoo P, Warburton D, Bellusci S (2007). Fgf10 dosage is critical for the amplification of epithelial cell progenitors and for the formation of multiple mesenchymal lineages during lung development. Dev Biol.

[18] Chao CM, El Agha E, Tiozzo C, Minoo P, Bellusci S (2015). A breath of fresh air on the mesenchyme: impact of impaired mesenchymal development on the pathogenesis of bronchopulmonary dysplasia. Front Med (Lausanne).

[19] Gupte VV, Ramasamy SK, Reddy R, Lee J, Weinreb PH, Violette SM, Guenther A, Warburton D, Driscoll B, Minoo P, Bellusci S (2009). Overexpression of fibroblast growth factor-10 during both inflammatory and fibrotic phases attenuates bleomycin-induced pulmonary fibrosis in mice. Am J Respir Crit Care Med.

[20] Volckaert T, Dill E, Campbell A, Tiozzo C, Majka S, Bellusci S, De Langhe SP (2011). Parabronchial smooth muscle constitutes an airway epithelial stem cell niche in the mouse lung after injury. J Clin Invest.

[21] Branchfield K, Li R, Lungova V, Verheyden JM, McCulley D, Sun X (2015). A three-dimensional study of alveologenesis in mouse lung. Dev Biol.

[22] Dickie R, Wang YT, Butler JP, Schulz H, Tsuda A (2008). Distribution and quantity of contractile tissue in postnatal development of rat alveolar interstitium. Anat Rec.

[23] Wendel DP, Taylor DG, Albertine KH, Keating MT, Li DY (2000). Impaired distal airway development in mice lacking elastin. Am J Respir Cell Mol Biol.

[24] Bostrom H, Willetts K, Pekny M, Leveen P, Lindahl P, Hedstrand H, Pekna M, Hellstrom M, Gebre-Medhin S, Schalling M, Nilsson M, Kurland S, Tornell J, Heath JK, Betsholtz C (1996). PDGF-A signaling is a critical event in lung alveolar myofibroblast development and alveogenesis. Cell.

[25] Perl AK, Gale E (2009). FGF signaling is required for myofibroblast differentiation during alveolar regeneration. Am J Physiol Lung Cell Mol Physiol.

[26] Chen L, Acciani T, Le Cras T, Lutzko C, Perl AK (2012). Dynamic regulation of platelet-derived growth factor receptor alpha expression in alveolar fibroblasts during realveolarization. Am J Respir Cell Mol Biol.

[27] De Langhe SP, Carraro G, Warburton D, Hajihosseini MK, Bellusci S (2006). Levels of mesenchymal FGFR2 signaling modulate smooth muscle progenitor cell commitment in the lung. Dev Biol.

[28] McGowan SE, McCoy DM (2013). Platelet-derived growth factor-A and sonic hedgehog signaling direct lung fibroblast precursors during alveolar septal formation. Am J Physiol Lung Cell Mol Physiol.

[29] Li C, Li M, Li S, Xing Y, Yang CY, Li A, Borok Z, De Langhe S, Minoo P (2015). Progenitors of secondary crest myofibroblasts are developmentally committed in early lung mesoderm. Stem Cells.

[30] Vaccaro C, Brody JS (1978). Ultrastructure of developing alveoli. I. The role of the interstitial fibroblast. Anat Rec.

[31] Torday J, Hua J, Slavin R (1995). Metabolism and fate of neutral lipids of fetal lung fibroblast origin. Biochim Biophys Acta.

[32] El Agha E, Herold S, Al Alam D, Quantius J, MacKenzie B, Carraro G, Moiseenko A, Chao CM, Minoo P, Seeger W, Bellusci S (2014). Fgf10-positive cells represent a progenitor cell population during lung development and postnatally. Development.

[33] Al Alam D, El Agha E, Sakurai R, Kheirollahi V, Moiseenko A, Danopoulos S, Shrestha A, Schmoldt C, Quantius J, Herold S, Chao CM, Tiozzo C, De Langhe S, Plikus MV, Thornton M, Grubbs B, Minoo P, Rehan VK, Bellusci S (2015). Evidence for the involvement of fibroblast growth factor 10 in lipofibroblast formation during embryonic lung development. Development.

[34] Barkauskas CE, Cronce MJ, Rackley CR, Bowie EJ, Keene DR, Stripp BR, Randell SH, Noble PW, Hogan BL (2013). Type 2 alveolar cells are stem cells in adult lung. J Clin Invest.

[35] Schultz CJ, Torres E, Londos C, Torday JS (2002). Role of adipocyte differentiation-related protein in surfactant phospholipid synthesis by type II cells. Am J Physiol Lung Cell Mol Physiol.

[36] Rehan VK, Torday JS (2012). PPARgamma signaling mediates the evolution, development, homeostasis, and repair of the lung. PPAR Res.

[37] Torday JS, Rehan VK (2002). Stretch-stimulated surfactant synthesis is coordinated by the paracrine actions of PTHrP and leptin. Am J Physiol Lung Cell Mol Physiol.

[38] Yamaguchi TP, Dumont DJ, Conlon RA, Breitman ML, Rossant J (1993). flk-1, an flt-related receptor tyrosine kinase is an early marker for endothelial cell precursors. Development.

[39] Peng T, Tian Y, Boogerd CJ, Lu MM, Kadzik RS, Stewart KM, Evans SM, Morrisey EE (2013). Coordination of heart and lung co-development by a multipotent cardiopulmonary progenitor. Nature.

[40] Greif DM, Kumar M, Lighthouse JK, Hum J, An A, Ding L, Red-Horse K, Espinoza FH, Olson L, Offermanns S, Krasnow MA (2012). Radial construction of an arterial wall. Dev Cell.

[41] Que J, Wilm B, Hasegawa H, Wang F, Bader D, Hogan BL (2008). Mesothelium contributes to vascular smooth muscle and mesenchyme during lung development. Proc Natl Acad Sci U S A.

[42] Havrilak JA, Shannon JM (2015). Branching of lung epithelium in vitro occurs in the absence of endothelial cells. Dev Dyn.

[43] Lazarus A, Del-Moral PM, Ilovich O, Mishani E, Warburton D, Keshet E (2011). A perfusion-independent role of blood vessels in determining branching stereotypy of lung airways. Development.

[44] Scott CL, Walker DJ, Cwiklinski E, Tait C, Tee AR, Land SC (2010). Control of HIF-1{alpha} and vascular signaling in fetal lung involves cross talk between mTORC1 and the FGF-10/FGFR2b/Spry2 airway branching periodicity clock. Am J Physiol Lung Cell Mol Physiol.

[45] Del Moral PM, Sala FG, Tefft D, Shi W, Keshet E, Bellusci S, Warburton D (2006). VEGF-A signaling through Flk-1 is a critical facilitator of early embryonic lung epithelial to endothelial crosstalk and branching morphogenesis. Dev Biol.

[46] DeLisser HM, Helmke BP, Cao G, Egan PM, Taichman D, Fehrenbach M, Zaman A, Cui Z, Mohan GS, Baldwin HS, Davies PF, Savani RC (2006). Loss of PECAM-1 function impairs alveolarization. J Biol Chem.

[47] Thebaud B, Ladha F, Michelakis ED, Sawicka M, Thurston G, Eaton F, Hashimoto K, Harry G, Haromy A, Korbutt G, Archer SL (2005). Vascular endothelial growth factor gene therapy increases survival, promotes lung angiogenesis, and prevents alveolar damage in hyperoxia-induced lung injury: evidence that angiogenesis participates in alveolarization. Circulation.

[48] Ding BS, Nolan DJ, Guo P, Babazadeh AO, Cao Z, Rosenwaks Z, Crystal RG, Simons M, Sato TN, Worgall S, Shido K, Rabbany SY, Rafii S (2011). Endothelial-derived angiocrine signals induce and sustain regenerative lung alveolarization. Cell.

[49] Entesarian M, Matsson H, Klar J, Bergendal B, Olson L, Arakaki R, Hayashi Y, Ohuchi H, Falahat B, Bolstad AI, Jonsson R, Wahren-Herlenius M, Dahl N (2005). Mutations in the gene encoding fibroblast growth factor 10 are associated with aplasia of lacrimal and salivary glands. Nat Genet.

[50] Rohmann E, Brunner HG, Kayserili H, Uyguner O, Nurnberg G, Lew ED, Dobbie A, Eswarakumar VP, Uzumcu A, Ulubil-Emeroglu M, Leroy JG, Li Y, Becker C, Lehnerdt K, Cremers CW, Yuksel-Apak M, Nurnberg P, Kubisch C, Schlessinger J, van Bokhoven H, Wollnik B (2006). Mutations in different components of FGF signaling in LADD syndrome. Nat Genet.

[51] Klar J, Blomstrand P, Brunmark C, Badhai J, Hakansson HF, Brange CS, Bergendal B, Dahl N (2011). Fibroblast growth factor 10 haploinsufficiency causes chronic obstructive pulmonary disease. J Med Genet.

[52] Klinger G, Levy I, Sirota L, Boyko V, Lerner-Geva L, Reichman B (2010). Outcome of early-onset sepsis in a national cohort of very low birth weight infants. Pediatrics.

[53] Carver BJ, Plosa EJ, Stinnett AM, Blackwell TS, Prince LS (2013). Interactions between NF-kappaB and SP3 connect inflammatory signaling with reduced FGF-10 expression. J Biol Chem.

[54] Benjamin JT, Smith RJ, Halloran BA, Day TJ, Kelly DR, Prince LS (2007). FGF-10 is decreased in bronchopulmonary dysplasia and suppressed by Toll-like receptor activation. Am J Physiol Lung Cell Mol Physiol.

[55] Noguchi A, Reddy R, Kursar JD, Parks WC, Mecham RP (1989). Smooth muscle isoactin and elastin in fetal bovine lung. Exp Lung Res.

